# Mr. Albert Corner

**Published:** 1911-05-06

**Authors:** 


### OBITUARY.

The death is reported at Westcliff of Mr. Albert Corner, M.R.C.S., L.R.C.P., at the age of forty-five. Mr. Corner was a student at St. Bartholomew's Hospital, and took his diploma in 1891. He had been senior clinical assistant at Moorfields Ophthalmic Hospital, and at the Ophthalmic, Throat, Nose and Ear Departments of St. Bartholomew's Hospital. He was an occasional contributor to the medical papers.

